# How Can Nanotechnology Help to Repair the Body? Advances in Cardiac, Skin, Bone, Cartilage and Nerve Tissue Regeneration

**DOI:** 10.3390/ma6041333

**Published:** 2013-03-28

**Authors:** Macarena Perán, María Angel García, Elena Lopez-Ruiz, Gema Jiménez, Juan Antonio Marchal

**Affiliations:** 1 Department of Health Sciences, University of Jaén, Campus Las Lagunillas, S/N, Jaén 23071, Spain; E-Mail: elruiz@ujaen.es; 2 Research Unit, University Hospital “Virgen de las Nieves”, Avda. de las Fuerzas Armadas, 2, Granada 18014, Spain; E-Mail: mangelgarcia@ugr.es; 3 Biopathology and Regenerative Medicine Institute (IBIMER), University of Granada, Avda. del Conocimiento S/N. CP Armilla, Granada 18100, Spain; E-Mail: gemajg@correo.ugr.es; 4 Department of Human Anatomy and Embryology, University of Granada, Avda. De Madrid, 11, Granada 18012, Spain

**Keywords:** nanotechnology, bio-scaffold, tissue engineering, nanostructures

## Abstract

Nanotechnologists have become involved in regenerative medicine *via* creation of biomaterials and nanostructures with potential clinical implications. Their aim is to develop systems that can mimic, reinforce or even create *in vivo* tissue repair strategies. In fact, in the last decade, important advances in the field of tissue engineering, cell therapy and cell delivery have already been achieved. In this review, we will delve into the latest research advances and discuss whether cell and/or tissue repair devices are a possibility. Focusing on the application of nanotechnology in tissue engineering research, this review highlights recent advances in the application of nano-engineered scaffolds designed to replace or restore the followed tissues: (i) skin; (ii) cartilage; (iii) bone; (iv) nerve; and (v) cardiac.

## 1. Introduction

The populations of developed countries are rapidly aging, causing an increment in age-related diseases like osteoarthritis, osteoporosis, or Parkinson’s disease. In addition to accidents, as well as poor health habits such as tobacco and stress, tissue or organ dysfunction can be provoked or even lost. To date, donor organ transplantation is the usual procedure to restore or enhance life expectancy. Nevertheless, this option is limited due to short supply and life-long immune suppression issues, as well as other associated side effects.

Tissue engineering, which is an alternative option for organ transplantation, is currently demonstrating great promise with first-in-man successful stories of tissue engineered implants [1]. This interdisciplinary science has as ultimate goal to design artifacts that (i) mimic natural tissues characteristics; (ii) fill up a space until the damage tissue is regenerated; (iii) temporarily replace tissue functions and; (iv) serve as a guide for tissue ingrowths. With this aim, nanotechnologists are applying their knowledge and experience using materials in a scale of less than 100 nanometers, to design and manufacture scaffolds that can replace the natural extra cellular matrix until host cells can repopulate and redo a new natural matrix. In addition, the biological substitutes should match the mechanical properties as the tissue is replacing to avoid mismatch between the synthetic graft and the surrounding native tissue. Furthermore, the materials used to construct the scaffold need to be biocompatible. The material must therefore be non-toxic, and its presence in the body should not elicit an immunological response. If the material used is biodegradable, its degradation kinetics should match the rate of tissue regeneration to ensure an optimal healing process. Other important parameters are adequate porosity to facilitate the delivery of nutrients to the regenerating cells, and appropriate nanotopography to promote cell adhesion and proliferation [2].

Natural or synthetic scaffolds have been tested in order to produce a clinically useful tissue scaffold of a target tissue or organ. Examples of natural scaffolds that have been applied clinically include decellularized dermis to treat burn injuries, as well as decellularized small intestine, ureter, or xenogeneic vessels to restore vascular function [3,4]. Although these materials have shown promising results in tissue repair, they have some drawbacks regarding mechanical properties, degradation, immunogenicity and cross-contamination. On the other hand, synthetic scaffolds have been constructed using synthetic materials or a combination between natural and synthetic materials and have demonstrated promising results in tissue repair [2]. The most commonly used natural biopolymers include demineralized bone matrix, agarose, collagen, hyaluronan, basement membrane, and alginate. Synthetic polymers include degradable polyesters, such as polyglycolic acid (PGA), polylactic acid (PLA), and their copolymers, poly (d,l-lactide-coglycolide) (PLGA). These biodegradable polymers have a long history of clinical use and are currently employed in various tissue engineering applications [5].

To construct suitable synthetic bio-scaffolds, the most widely chosen technique is electrospinning. This method allows the production of nanofibrous scaffolds with specific and desired properties and functionality. Importantly, nanofibrous scaffolds possess an extremely high surface-to-volume ratio, tunable porosity, and malleability to conform to a wide variety of sizes and shapes with a desirable 3D pattern [6].

**Figure 1:**
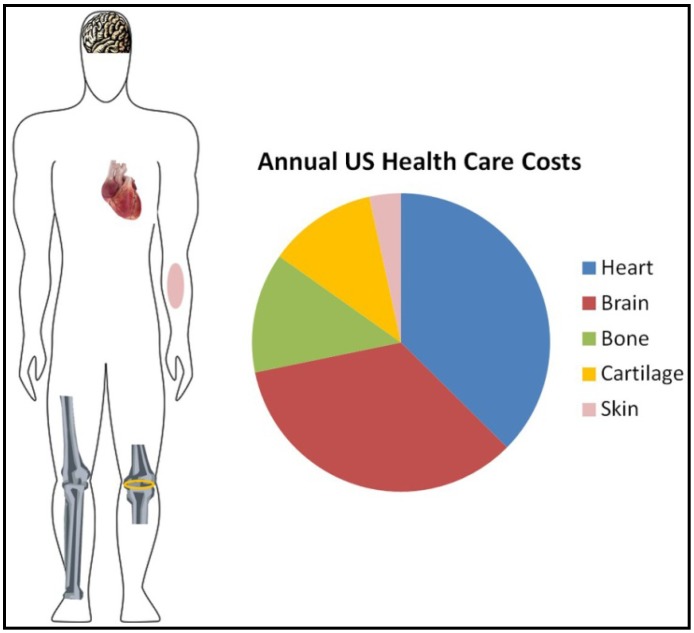
Representative figure showing the annual cost in the United Stated of America for main diseases related to tissue degeneration. The cost is shown in billions of US dollars.

In this work we have focused our attention on five tissues whose degeneration or dysfunction lead to chronic health problems and cause a steep increase in health care costs (Figure 1).

In the United States, chronic wounds affect around 6.5 million patients and an excess of $25 billion is spent annually [7]. In addition, the direct and indirect health care costs associated with all forms of arthritis is approximately 86 billion dollars per year [8]. Furthermore, nearly $95 billion of health care dollars are used annually to treat patients with osteoporosis-related fractures, excluding the expenses caused by fractures in healthy young people from accidents [9]. The costs increase significantly when it comes to related brain diseases (such as Alzheimer’s disease; blindness, deafness, brain injury; epilepsy, multiple sclerosis, Parkinson’s disease, spinal cord injury, or stroke) with an estimated cost each year of $273 billion [10]. Finally, cardiovascular diseases have an overall cost per year of $273 billion [11].

This review provides an overview of the progress of nanotectnology application in tissue engineering research, highlighting recent advances in the application of nano-engineered scaffolds designed to replace or restore tissues like (i) skin; (ii) cartilage; (iii) bone; (iv) nerve; and (v) cardiac.

## 2. Electrospinning

Polymeric nanofibers can be processed by a number of techniques such as drawing, template synthesis, phase separation, self-assembly and electrospinning. Among these methods, the most successful for tissue engineering applications is the electrospinning process. The main advantage of this method is that electrospun scaffolds can be characterized by a complex micro-scale structure responsible for its macroscopic mechanical behavior. In this sense, various parameters can be controlled in the process of nanofiber creation: (i) polymer solution parameters (viscosity, surface tension, conductivity, *etc.*); (ii) electrospinning process parameters (voltage, federate, tip-to-collector distance, *etc.*); and, (iii) ambient conditions (humidity). This allows the creation of various types of nanofibers with different thickness, pattern and forms which can be used to create various types of scaffolds [12].

**Figure 2:**
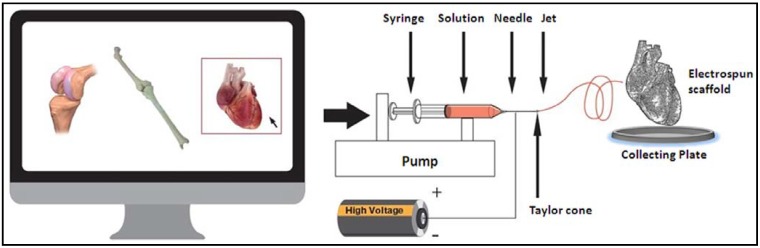
Schematic diagram of the electrospinning process showing a glass syringe containing polymer solution; a nanofiber jet; a copper collecting plate and a power supply.

In brief, electrospinning consists of a pipette to hold the polymer solution (spinneret), two electrodes, a high voltage power supply and a grounded collecting plate (usually a metal screen, plate, or rotating mandrel) (Figure 2). The production of polymer filaments is done by the electrostatic force, through the electrically charged jet of polymer solution. The polymer drops from the tip of the pipette and is drawn into a fiber due to the high voltage. The jet is electrically charged and the nanofibers are formed by the narrowing of the ejected jet stream as it undergoes increasing surface charge density due to evaporation of the solvent. The fiber is then collected as a mesh of fibers on the surface of a grounded target, the collecting plate, thus forming the electrospun scaffold [6]. A very important feature of this technique is that it provides a large surface area-to-volume ratio, which facilitates cellular uptake and nutrient diffusion. In addition, the fiber diameters can be controlled to mimic the extracellular matrix (ECM) fibrous architecture [13]. Moreover, electrospinning produces nanoarchitecturely patterned fibers in random or aligned form, which greatly influence the cell orientation and function [14,15].

Since the physical and biological properties of the electrospun nanofibrous scaffolds—including hydrophilicity, mechanical modulus and strength, biodegradability, biocompatibility, and specific cell interactions—can be controlled, the biomedical applications of functional nanofibrous scaffolds have great potential. Those biological parameters are determined by the polymer chemical compositions. Playing with polymer physics, copolymerization and polymer blending to combine different polymers can yield new material properties. In addition, the performance of the electrospun scaffold can be further controlled by adjusting the diameter and morphology of the nanofibers, desirable 3-D patterns (e.g., layered structures) and the porosity through the electrospinning processing technology. Thus, by selecting a combination of proper components and by adjusting the component ratio, properties of electrospun scaffolds can be tailored with desired new functions [6]. Technological advances could lead to the development of a versatile electrospinning system able to create different tissues scaffold (Figure 1).

## 3. Skin Regeneration by Nanotechnological Approaches

The primary function of the skin is to act as a barrier; consequently, any related problem such as burns, chronic wound, ulcers or accidents can cause serious health complications. The apparently simple structure of the skin, consisting of two layers, the epidermis and the dermis, and its easy target localization, has encouraged the search for therapeutic alternatives. In this regard, nanobiotechnology emerges as a promising hope to improve wound healing and skin restoration.

Skin tissue engineering is based in the creation of scaffolds that must share the followed minimal characteristics: (i) biocompatibility; (ii) support for cell attachment and proliferation; and, (iii) to imitate the ECM as closely as possible [16,17]. One of the main difficulties found in the application of this artificial skin is the problems related to adhesion and integration of the scaffolds to the topography of the wound, while maintaining physical and mechanical properties [18,19].

Electrospun nanofibrous scaffolds have been created to mimic the three-dimensional fiber network of the collagen fibrous structure. They are composed of collagen fibers that are formed hierarchically by nanometer-scale multi-fibrils and have been proved to support cell adhesion, proliferation, and differentiation mimicking the fibrous architecture of the ECM [5,20]. In addition, the electrospinning technique allows for control over the desired pore diameter, distribution, total volume, total area, and, consequently, the final porosity of the structure. A pioneering *in vivo* study has shown the benefits of covering wounds with polyurethane membranes produced via electrospinning. These membranes increased the epithelialization rate and formed a well-organized dermis [21]. The electrospun nanofibrous membrane could control water loss by evaporation, was permeable to oxygen, and promoted fluid drainage ability, while inhibited exogenous microorganism invasion. Other examples of good antibacterial activity was shown using collagen/chitosan-immobilized polypropylene wound-dressing membranes, demonstrating an excellent remodeling effect after histological examination with respect to the construction of vein, epidermis, and dermis at 21 days after skin injury [22].

**Figure 3:**
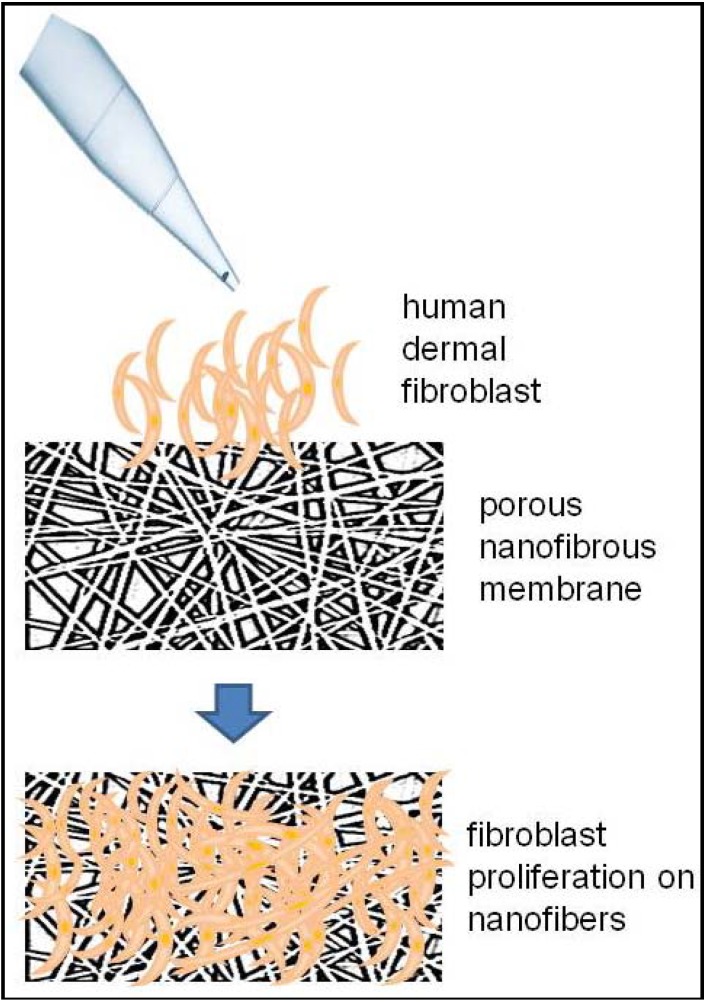
Human dermal fibroblast can be cultured on electrospun nanofibrous membrane to create *in vitro* allogeneic dermal substitutes.

Fibroblasts are the cell type best indicated for wound healing proposes [23]. In fact, seeding fibroblasts into dermal substitutes have been shown to improve wound healing [24]. In this respect, poly(l-lactic acid)-*co*-poly(ε-caprolactone) and gelatin (PLACL-G-P) nanofibrous scaffolds provided enough space for fibroblast ingrowth and induced the formation of a dermal substitute [25]. Figure 3 shows a schematic example of an electrospun-scaffold for the wound-healing proposes. In addition, the incorporation of collagen to the polycaprolactone-nanofibrous membrane improved attachment and proliferation of fibroblast [26]. Other studies have used a scaffold with mesenchymal stem cells (MSCs), and found an increased proliferation rate and differentiation of MSCs when combining poly (l-lactic acid) poly-*co*-(3-caprolactone) with a biomaterial [27]. In another assay, three-dimensional chitosan nanofibers were implanted in mice to cover full-thickness skin wounds and were able to induce a faster regeneration of both the epidermis and dermis compartments when compared to other structures such as sponges [28].

The potential clinical application of exogenous growth factors to chronic wounds, in an effort to accelerate healing, is not new, and in fact dates back more than 10 years [29]. Recently, electrospun fibers with a core-sheath structure and loaded with basic fibroblast growth factor-encoding plasmid were found in diabetic mice. The gradual growth factor released revealed significantly higher wound recovery rate with improved vascularization, enhanced collagen deposition and maturation, complete re-epithelialization and formation of skin appendages [30].

**Figure 4:**
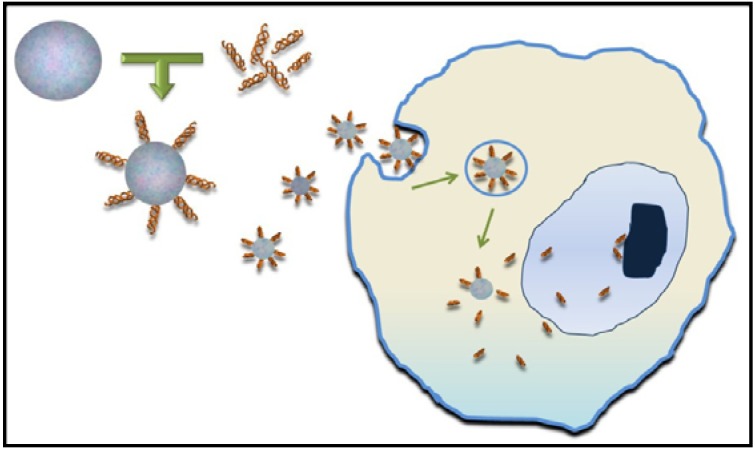
Synthetic nanoparticles are able to conjugate peptides, growth factors, nitric oxide or other molecules onto the particle surface and act as delivery vehicles.

The development of nanotechnology has also allowed the creation of nanoparticles (NPs) that act as a vehicle and carrier of biological factors that induce skin regeneration (Figure 4). In fact, several promising results have been obtained in studies using NP bearing: growth factors, thrombin, nitric oxide, opioids or protease inhibitors [31]. For example, thrombin-conjugated iron oxide NPs improved tensile strength of the wounds, thereby indicating a significant acceleration of the healing process [32].

## 4. Nanotechnological Advances in Cartilage Repair

Cartilage injuries lead to joint pain and loss of function. Mature hyaline cartilage has a very low self-repair potential due to its intrinsic properties. For this reason, researchers have focused in the search of methods to reproduce the tissue characteristics of hyaline cartilage and induce complete cartilage repair. A new approach for the treatment of articular cartilage defects is the use of biocompatible scaffolds [33]. There are plenty of polymers, but only some of them are suitable for cartilage tissue engineering. Natural materials used in the field of cartilage engineering include alginate, agarose, chitosan, chondroitin sulfate, hyaluronan, collagen, fibrin gelatine and silk fibroin. It has been demonstrated that these natural material could potentiate the production of collagen type II and sulfated glycosaminoglycans by both chondrocytes and stem cells [34]. Nevertheless, despite their biocompatibility, their potential for clinical use is limited by poor mechanical strength, immunogenicity and their rapid degradation upon implantation if not are cross-linked with appropriate chemical reagents [35]. Instead, synthetic materials present more easy molding characteristics, relatively easy production and the ability to control dissolution and degradation [36]. The synthetic materials most widely used are poly (a-hydroxy acids), especially poly (lactic acid) (PLA), poly (glycolic acid) (PGA) and their co-polymers (PLGA), poly (ε-caprolactone) (PCL), poly(propylene fumarate) and polyethylene glycol (PEG) [37,38].

ECM of cartilage tissue is comprised of collagen and proteoglycans which are nanometers in scale. Thus scaffolds for cartilage tissue engineering have to be accomplished on the nanoscale to achieve similar mechanical and physical properties to native tissue. To better recapitulate the ECM environment for cartilage tissue engineering, researchers have introduced several biological signals, including chondroitin sulfate (CS), hyaluronic acid and collagen, into tissue-engineered scaffolds. Recently, nanofibrous scaffolds composed of poly (vinyl alcohol) (PVA), a hydrophilic synthetic biodegradable polymer, and chondroitin sulfate, have been shown to enhance tissue formation *in vitro* and also *in vivo*, when these were implanted into rat osteochondral defects [39]. In addition, combination of PVA-PCL electrospun nanofiber scaffolds with MSCs showed improvement on tissue healing compared with those which received cell-free scaffolds, suggesting their potential as a suitable graft for articular cartilage reconstruction [40].

Another strategy for cartilage tissue engineering is to induce chondrogenic differentiation of adult stem cells by the delivery of growth factors included in nanoparticles that are embedded into the scaffolds. For example the continuous and controlled release of TGF-β1 from a heparin-functionalized NP within a fibrin/PLCL scaffold was proved to enhance and maintain chondrogenic differentiation of implanted cells [41]. In addition, biodegradable PLGA NPs have been used as gene delivery vehicles to induce chondrogenesis in hMSCs [42,43]. Recently, Jeon *et al.* pretreated PLGA NPs with PEI to modify the particle surface and conjugate SOX9 and a small interfering RNA of the Cbfa-1 gene expressed during osteogenesis [44]. *In vivo* results of injected MSCs that were encapsulated in fibrin hydrogels and transfected with PEI/SOX9 plus a Cbfa-1 siRNA showed a markedly increased expression of genes associated with chondrogenesis, whereas genes and proteins associated with osteoblasts did not show the same expression increment [44]. Others studies also demonstrate that combination of hMSCs, encapsulated in fibrin hydrogel, and NPs containing a specific growth factor represent a suitable niche for the differentiation of transplanted hMSCs [45,46]. Furthermore, scaffolds can be chemically modified to contain bioactive molecules such as peptides or heparin for creating a better microenvironment of cell adhesion and growth for use in cell therapy applications [41,47,48,49].

Surface modifications allow introducing nanofeatures into the scaffolds. For example, surface modifications with nano-hydroxyapatite (NHA) have been demonstrated to facilitate and promote cartilage regeneration. Actually, a PLGA/NHA scaffold seeded with MSCs was evaluated in a rat model. After implantation, articular osteochondral defects were filled up with smooth and hyaline-like cartilage with abundant glycosaminoglycan and collagen type II deposition, but deficient in collagen type I [50].

In addition, for the repair of osteochondral units, several authors have highlighted the need for biphasic scaffolds to reproduce the osteocartilaginous anatomical structure. In fact, a bilayer porous PLGA calcium-sulfate biopolymer (TruFit) is largely commercialized for clinical application. Although preclinical experimentation is promising [32], information on the long-term durability is still not available.

The safety and performance of the newly developed type I collagen hydroxyapatite nanostructured biomimetic osteochondral scaffold have been tested in a pilot clinical study. Clinical evaluation by magnetic resonance imaging showed that this scaffold promoted bone and cartilage tissue restoration [51].

Even though later advances in scaffold design for cartilage repair are relevant, improvement of mechanical strength, cell adherence, viability and metabolism of the neocartilage constructs are still necessary for their translation into clinical use.

## 5. Applying Nanotechnology to Bone Reconstruction

Trauma, pathological degeneration or congenital deformities make bone one of the most commonly transplanted tissues worldwide [52,53,54]. Autologous bone grafting and bone allografts are the usual treatment for reconstruction of skeletal defects. However, open surgery involves a considerable risk of morbidity and implant failure in patient population. As a result of these limitations, the engineering of new bone to replace the damaged bone based on synthetic biomaterials such as metals, polymers, porous ceramics, hydroxyapatite, collagen sponges or hydrogels, among others, have been developed in the past few years [55].

Despite substantial progress, the construction of structures able to provide the suitable physical and biological properties of the bone still presents challenges. Bone is comprised of hierarchically arranged collagen fibrils, hydroxyapatite and proteoglycans [56]. To mimic the natural bone nanocomposite architecture, novel biomaterials and nanofabrication techniques are currently being employed and many different nanostructures have already been designed and tested. Electrospinning has been extensively applied to create bone nanofiber scaffolds and biomaterials typically used for this purpose, including synthetic organic polymers such as PCL [57], PLGA, PLLA and natural polymers, such as chitosan [58] and silk fibroin [59]. The combination of synthetic and natural materials has also been studied, in fact, electrospun poly(l-lactic acid)/collagen nanofibrous scaffold have been shown to significantly induce osteogenic differentiation of human MSCs and the formation of bone minerals [60]. Recently, bioactive macromolecules like poly-benzyl-l-glutamate and nanohydroxyapatite have been introduced on the surface of polymeric nanofibers, and were proven to regulate and improve specific biological functions like adhesion, proliferation and differentiation of adipose-derived stem cells [61].

Among the materials used for bone-reconstruction, PLLA is a biocompatible polymer with the advantage of being highly biodegradable. For this reason, PLLA have received the approval of the Food and Drug Administration (FDA) to be use in bone reconstructive surgery [62]. PLLA nanofibers are often functionalized to improve their biological performance with peptides such as RGD (Arg-Gly-Asp); with osteogenic molecules such as hydroxyapatite; or with proteins such as collagen and the growth factor bone morphogenic protein 2 (BMP-2) [63]. In fact, direct incorporation of BMP-2 into PLLA nanofibers enhances the osteoinductivity of the scaffolds. It has been shown that PLLA nanofibers facilitate colonization of bone defects and in combination with BMP-2 increase bone generation, PLLA/BMP-2 implants being able to close critically sized calvarial defects within eight weeks [62].

Current orthopedic implants fail in an appropriate osteo-integration limiting implant lifespan. Recent studies are focused in altering the surface topography of materials at nanoscale level to secure integration with the surrounding tissue and to avoid extrusion and movement. Indeed, nanotopography has been shown to influence the type, quantity and conformation of adsorbed protein, and control cellular adhesion to the surface [64]. Titanium, as a biocompatible material, has been used to enhance implant incorporation in bone for dental, craniofacial, and orthopedic applications. Studies have demonstrated that nanoporous titanium dioxide (TiO_2_) surface modification alters nanoscale topography improving soft tissue attachment on titanium implants surface [65,66]. For example, the uses of nanoporous TiO_2_ surface-modified implants, in a human dental clinical study, showed that TiO_2_ thin film increased adherence in early healing of the human oral mucosa and reduced marginal bone resorption [67].

Nanostructured implant surfaces are also known to enhance osteoblast activity. Using a hydrothermal technique, a simple one-step wet chemical method, non-periodic nanostructures have been developed to surface modify metallic titanium implants. Among the nanomorphologies tested, the nanoleafy pattern showed the strongest influence on protein adsorption, *in vitro* osteoblast cell proliferation and differentiation. *In vivo*, these nanostructures have also demonstrated a higher percentage of bone contact without producing any inflammatory response. These results point to the importance of specific nanomorphologies in controlling tissue integration [68].

In another study, the effect on osteoblast differentiation of TiO_2_ nanofiber meshes, fabricated using an electrospinning method to create different surface micro-roughness and nanofiber diameters, was evaluated. Osteoblast differentiation and local factor production were regulated by both roughness surface and the nanotopography, indicating that scaffold structural characteristics alone can be used to drive cell differentiation and create an osteogenic environment without the use of exogenous factors [69].

Nanotube structures have also been shown to have great potential for bone regeneration; their shape is very similar to that of the nanofibers with the only difference that the nanotubes are hollow. Bioactive helical rosette nanotubes are self-assembled nanomaterials, formed in water from synthetic DNA base analogs that mimic the helical nanostructure of collagen in bone. This technology has been used to create a biomimetic nanocomposite combined with nanocrystalline hydroxyapatite, and biocompatible hydrogels which increased osteoblast adhesion [70]. Rosette nanotubes have also been incorporated into various natural and synthetic biomaterial scaffolds. For example, combination of hydrogels, specifically poly(2-hydroxyethyl methacrylate) (pHEMA) and hydroxyapatite (HA) NP, have been demonstrated to improve long-term functions of osteoblasts by increasing the collagen synthesis, alkaline phosphatase activity, and calcium deposition [71].

Carbon nanotubes (CNTs) are other suitable scaffold materials that have proved to support osteoblast proliferation. Indeed, CNTs possess exceptional mechanical, thermal, and electrical properties, facilitating their use as reinforcements or, in combination with other biomaterials, to improve and to support bone growth [72,73]. In fact, Li *et al.* have showed that CNTs could induce ectopic bone formation in the dorsal musculature of mice, suggesting that these nanotubes might not only improve cell attachment and proliferation but also differentiate the inducible cells derived from soft tissues to osteogenic cells [65].

Clinical therapies implying the use of nanotechnology in bone regeneration are still in the beginning stages. Considering that hydroxyapatite is one of the major components in the bone matrix, synthetic nanocrystalline HA has been used to construct scaffold for bone substitutes. Recently, the bone healing ability of a nanocomposite (DBSint^®^), approved for clinical use, constituted by biomimetic nanostructured Mg-hydroxyapatite and human demineralized bone matrix has been investigated. The clinical-radiographic and histomorphometry study in subjects undergoing high tibial osteotomy, demonstrated that these nanocomposites are safe and effective. However, non-reabsorbed graft remnants surrounded by soft tissues were observed, thus the benefits of this approach in a long-term outcome are still unclear and require further investigation [74]. Schwarz *et al.* undertook a four-year study of patients treated of moderate intrabony peri-implantitis defects using either a nanocrystalline hydroxyapatite or a natural bone mineral (BioOsss spongiosa granules) in combination with a collagen membrane (BioGides) and found bone reconstruction [75].

## 6. Nanotechnology for Nerve Regeneration

Incomplete recovery from peripheral nerve injuries can produce a diversity of negative outcomes, including numbness, impairment of sensory or motor function, the possibility of developing chronic pain, and devastating permanent disability. The gold standard treatment is surgery, which requires an autologous nerve graft. Nevertheless, many complications are related to this technique including the sacrifice of the donor nerve function, limited availability of donor tissue, and formation of potentially painful neuromas. Consequently, it is needed to develop new strategies to create nerve artificial prosthesis to solve nerve donor-associated complications.

One of the biggest challenges in peripheral nerve tissue engineering is to create an artificial nerve graft that could mimic the ECM and assist in nerve regeneration. Bio-composite nanofibrous scaffolds made from synthetic and natural polymeric blends provide suitable substrate for tissue engineering and it can be used as nerve guides eliminating the need for autologous nerve grafts. Nanotopography or orientation of the fibers within the scaffolds greatly influences the nerve cell morphology and outgrowth, and the alignment of the fibers ensures better contact guidance of the cells. A bioartificial nerve conduit must meet the overall requirements of a suitable bio-scaffold; it must therefore be biodegradable, biocompatible and non-immunogenic. Furthermore, nerve conduits should also be engineered to achieve specific characteristics such as to possess an adequate tensile strength without compromising flexibility [76].

It is essential to select the appropriate material in order to reproduce the specific characteristic of the native nerve. In this respect, numerous materials, synthetic and natural, have been tested for manufacturing nerve conduits.

Aliphatic polyesters are biocompatible polymers that can be synthesized into fibers via electrospinning [76]. Actually, several PGA and PCL nerve guidance conduits have been approved by the FDA for clinical use in peripheral nerve repair [77]. A successful example is Neurotube^®^, an absorbable woven PGA mesh tube designed for peripheral nerve repair or reconstruction (Synovis Micro Companies Alliance, Birmingham, AL). The efficacy of this scaffold has been demonstrated by a large multicenter clinical trial, reporting very encouraging results for digital nerve reconstruction [78]. Further clinical reports investigating the Neurotube^®^ conduit in motor reconstruction of the spinal accessory nerve, several facial nerves and forearm median nerves showed promising clinical outcomes. Nevertheless, although Neurotube^®^ is the preferred synthetic nerve conduit among surgeons, there are still some issues that decrease their efficiency, such as the high rate of degradation that reduces its mechanical properties, and the formation of acidic degradation deposits.

To solve these limitations and to improve efficacy, more recently, numerous PGA derivative compounds and combinations have begun to emerge. For example, neural stem cells and Schwann cells have been cultured in combined PLGA conduits and neurotrophin-3 (NT-3) to generate NT-3-loaded PLGA carriers *in vitro* [79]. In addition, the incorporation of growth factor-microspheres or VEGF-microspheres to PLGA has proved to increase nerve formation when grafted to a rat model [80]. Another study showed nerve regeneration after two months of PLGA tube, containing rat dental pulp cells embedded into a collagen gel, transplantation [81]. Finally, introduction of autologous MSCs to PLGA scaffolds improved the repair and rehabilitation of a large gap after peripheral nerve injury in dogs [82] and rats [83].

Another biocompatible polymer that has gained considerable interest in nerve regeneration research field is PCL. The main advantages of PCL are that can be easily manipulated with low processing costs. Its high processability is attributed to the fact that PCL is very soluble in a wide range of organic solvents and, moreover, its crystalline nature enables easy formability at relatively low temperatures. Its degradation products are less acidic than PLA and non-toxic, which causes less damage to the surrounding tissue environment and do not trigger an inflammatory response [84]. Neurolac^®^ (Polyganics Inc., the Netherlands) is a PCL nerve conduit that has also been approved by the FDA. A randomized clinical trial, with 30 patients suffering from hand nerve lesions, has been conducted to test the nerve reconstruction capacity of Neurolac^®^. Patients were randomized for treatment either with autologous nerve grafts or with Neurolac^®^. Although some complications were reported for the Neurolac®-treated group, none were directly related to the use of the PLC device. Interestingly, the recovery of sensibility between groups was comparable [85], however Neurolac^®^ showed some limitation, principally, its high rigidity [83].

Another strategy for nerve regeneration is the combination of PCL devices with cells or natural materials. For instance, MSCs grown in the nerve scaffold has been proved to improve nerve regeneration after a nerve transection in mice [86]. On the other hand, collagen/PCL fibrous scaffold successfully supported nerve regeneration through an 8-mm sciatic nerve gap in adult rats, achieving similar electrophysiological and muscle reinnervation results as autografts [87].

Peptide amphiphiles (PAs) are peptides with the ability to form spontaneous self-assembly nanofibers and a dual functionality of simultaneously being hydrophobic and hydrophilic. This balance of polarity between attractive and repulsive forces within the nanomolecular construct further alludes to their novel properties [76]. In fact, the nanofiber self-assembly framework of PAs has proved to promote the migration and proliferation of neural cells [88]. PAs can also function as efficient drug and gene delivery platforms. Actually, various therapeutic agents can be incorporated into PAs to augment the recovery process and minimize immune response. Some examples of PAs, with potential to be a candidate of nerve conduits, is IKVAV, a pentapeptide, made up of a sequence of amino acids Ile-Lys-Val-Ala-Val, first identified in the A chain of laminin. IKVAV is a neurite-promoting laminin epitope, and it has been demonstrated to upregulate the proliferation of neural cells [76]. Another is RGD (Arg-Gly-Asp) a tripeptide able to mediate peripheral neuron regeneration, in fact integration of RGD into PAs promoted cell proliferation and differentiation [89]. A plethora of peptide sequences can also be incorporated into PAs, making these nanofibers extremely versatile and customizable [76].

Natural materials offer increased levels of biocompatibility, decreased toxicity and enhanced migration of support cells when compared with synthetic materials. Collagen has widespread use as a biological material including peripheral nerve repair, because, when purified, it is weakly antigenic. Diffusion processes through collagen matrices are facilitated by its smooth microgeometry and transmural permeability. In addition, the adhesive property of collagen for different cell types also permits enhanced survival and proliferation [84]. NeuraGen^®^ (Integra Life Sciences Corporation, Plainsboro, NJ, USA) was the first semi-permeable Type I collagen nerve guidance conduit to receive approval from the FDA. The clinical experience using NeuraGen^®^ was reported by Taras *et al.* in a medical study on peripheral nerve reconstruction [90]. Patients tolerated splinting and resisted exercise without negative clinical consequences. In another study, NeuraGen^®^ has been compared with direct suture repair, in patients with complete traumatic nerve injuries. Results showed that patients who received NeuraGen^®^ had lower post-operative pain than those treated with direct suture repair. The overall study conclusion was that entubulation nerve repair using the NeuraGen^®^ is as effective method of joining severed nerves as direct microsurgical suture for short gap graft repair (data presented in the American Society for Peripheral Nerve) (revised in Kehoe *et al.*) [84]. To improve NeuraGen’s^®^ limitations, such as the long duration of its biodegradation, other collage Type I derivate structures have been designed;however, no conclusive clinical data have yet been documented [84].

Recently, composite materials based on the coupling of conductive organic polymers and carbon nanotubes have shown to possess properties of the individual components and the benefit of a synergistic effect. For instance, multi-wall carbon nanotube (MWCNT)/polymer composites are hybrid materials that combine numerous mechanical, electrical and chemical properties and can be used for the development of nerve guidance channels to promote nerve regeneration. This biomaterial is a suitable substrate that increases electronic interfacing between neurons and can be employed to create micro-machined electrodes with potential applications in neural regeneration, prosthetic devices and brain implants [91].

The use of micro-electromechanical systems stimulation, through modulation of ions around the nerve, is a novel nanotechnology strategy for modulating nerve impulse activation. These findings have potentially significant implications for the design of special nano-enhanced materials that could be used to promote nerve regeneration and rehabilitation [92].

Bridging larger nerve gaps between proximal and distal ends requires exogenous tubular constructs with uniaxially aligned topographical cues to promote the axonal regrowth. In this respect, electrospun nanofibrous scaffolds are a good candidate to fill up the gap of the injured nerve. Recently, it has been demonstrated that the alignment of nanofibers has a significant influence on the adhesion and proliferation of Schwann cells. The axially aligned nanofibers were shown to mimic the fibrin cable architecture and, thereby, this approach may represent an ideal scaffold for extending the growth of axonal processes [93].

Additionally, flexible nerve agent sensors, based on hydroxylated poly(3,4-ethylenedioxythiophene) nanotubes with surface substructures such as nanonodules and nanorods have been explored. The surface substructures can be grown on a nanofiber surface by controlling critical synthetic conditions during vapor deposition polymerization on the polymer nanotemplate, leading to the formation of multidimensional conducting polymer nanostructures where hydroxyl groups are found to interact with the nerve agents. Representatively, the sensing response of dimethyl methylphosphonate, as a simulant for sarin, is highly sensitive and reversible from the aligned nanotubes and the sensor has excellent mechanical bendability and durability [94].

## 7. Nanotecnology for Cardiac Tissue Regeneration

Heart stroke and valvular heart disease are a significant cause of morbidity and mortality worldwide. Myocardial infarction results in reduced cardiac function due to cardiomyocyte death. As the proliferative potential of the terminally differentiated cardiomyocytes is low, the heart is unable to repair itself and, after damage, non-functional scar tissue is formed. On the other hand, damage or defect in one of the four heart valves can ultimately lead to heart failure. Classical replacement surgery involves the implantation of mechanical valves or biological valves (xeno- or homografts).

Engineering the heart represents a real challenge for a new branch of multidisciplinary researchers whose goal is regenerating the damaged cardiac tissue. Certainly, it is not a simple matter of patching the damaged tissue. The elasticity and contractive properties of this perfect pump have to be guaranteed in order to avoid complications, such as arrhythmias or dysfunction, which could prevent the correct impulsion of blood to the entire body [95,96].

Cell injection directly into the heart has proven to revolutionize the treatment of heart disease [97,98]. Some clinical trials have been conducted injecting autologous stem cells derived from bone marrow, and some benefits such as improvement in left ventricular ejection fraction and concomitant increase in myocardial perfusion have been proven [99,100,101]. Moreover, enhancement in myocardial oxygen consumption [102] and in the contractility properties of the scarred area has been demonstrated [103]. A recent meta-analysis performed to evaluate the effectiveness of adult bone marrow-derived stem cell injection to treat acute myocardial infarction concluded that a moderate and significant improvement in global heart function was achieved after the stem cell therapy [104]. However, although beneficial, the effects of stem/progenitor cell administration on cardiac function in the clinical setting have not quite fulfilled expectations [102].

Considering the drawbacks of cell implantation and the fact that many cardiovascular diseases can lead to heart damage from the necessarily replaced functional structures of the heart—such as valves, or even the whole heart—there is a great need for approaches that create cardiac tissue via bioengineering. Advances in nanotechnology have allowed researchers to fabricate scaffolds with the aim to mimic the natural cell environment with the physical properties that influence the physiological behavior of the tissue. Thus far, contractile cardiac grafts have been created *in vitro* and are postulated as a system for replacing infarcted myocardium and to enhance cardiac function. Furthermore, tissue engineering of heart valves or injection of nanomaterials to improve the function of faulty heart valves, are newly emerging alternatives that improve current modes of therapy in valvular heart surgery.

The use of a degradable, nanofibrous scaffold made by electrostatic fiber spinning have been postulated to be a feasible method to produce cardiac grafts with clinically relevant dimensions [105]. A variety of biomaterials have been tested, alone or in combinations, to fulfill the requirements of myocardial regeneration. The priority is to find polymers with specific elastic and ductile mechanical properties that can, for example, mimic the necessary anisotropy of cardiac tissue with the combination of PCL and gelatin, and be properly oriented, yield excellent results and improve adhesion and alignment of cardiomyocytes in the nanofibrous mesh [106]. A very interesting study shows that nanofibrous scaffolds made of L-lactic acid with trimethylene carbonate (P(L)LA-*co*-TMC) promote cardiomyocyte proliferation and efficiently preserve cell morphology, without hampering expression of sarcomeric alpha actinin marker, thus demonstrating its potential as a synthetic biomaterial for myocardial tissue engineering [107].

Furthermore, a recent study revealed the proper ratio of poly(1,8-octanediol-*co*-citrate) (POC) and poly(l-lactic acid)-*co*-poly-(3-caprolactone) (PLCL) to obtain a nanoscaffold with mechanical properties, such as elasticity and tensile strength, similar to the cardiac tissue [96,108].

Going even further, researchers have developed a biocompatible scaffold that not only has good physical, chemical and mechanical properties, but also present the ability to differentiate cells to cardiomyocytes. In this respect, Gupta *et al.* have created a scaffold combining PEG-PCL-CPCL with an inhibitor of bone morphogenetic protein (BMP) which promotes differentiation of ESCs toward functional cardiomyocytes [109]. In addition, Sreerekha *et al.* showed that a scaffold that combines fibrin and PLGA was able to stimulate cardiomyocyte MSC differentiation [110]. Other studies have used a chitosan nanoscaffold coated with fibronectin and proposed a cardiomyocyte-fibroblast co-culture system that resulted in a cardiac tissue-like structure, where cardiomyocytes maintained their morphology and polarity and contracted synchronously [111]. Recently, cardiac patches of PEG nanoscaffold, embedded in a fibrin hydrogel together with cardiac progenitor cells, were implanted in the ventricle wall of a rat infarction model. The engraftment improves the infarcted area, increasing cell viability and ECM collagen organization [112].

Another nanotechnological variant consists in the use of NPs, which present important advantages for a targeted therapy. For instance NPs can easily cross the endothelium, and can be administrated by a non-invasive procedure, intravenously or by inhalation [113,114]. Numerous *in vivo* studies have illustrated the advantages of the use of NPs as complementary therapy in cardiovascular diseases. For example, phosphatidylserine liposomes administered intravenously to a myocardial infarction mouse model, proved to be capable of promoting angiogenesis, remodeling and to prevent ventricular dilatation [115]. In addition, NPs have been employed to allow factor and/or cytokine administration. Anti-P-selectin-conjugated liposomes containing VEGF, improved cardiac function and vascular structure in mice after myocardial infarction [116].

Actually, the two main nanotechnological strategies for heart valve disease treatment are: (i) the use of tissue engineering to produce fully functional heart valve; or (ii) the employment of NPs to alter the physical structure and behavior of faulty valves.

Biological valves are used for valve replacement in surgical therapy for end-stage valvular diseases. But biological prostheses lead to complications such as limited lifespan of the implant due to deterioration and calcification of the valve structure, together with problems related to immunological response [117]. Nano-engineered heart valves are a promising approach to overcome the limitations of conventional heart valve prostheses. In fact, these valves have been tested in animal models showing excellent tissue remodeling. For instance, fibrin scaffold in combination with arterial-derived cells were inserted in a sheep model and, after three months, the fibrin scaffold was replaced by new tissue containing mature collagen along with functional blood vessels [118]. Another successful example of a nano-engineered heart valve has been shown by Kalfa *et al.*, who used polydioxanone (PDO) and the electrospun technique to construct bioabsorbable valved patches that supported MSCs growth [119]. After eight months of implantation in the heart of growing lambs, the PDO scaffold was completely degraded and replaced by a viable, three-layered, endothelialized tissue.

One important aspect that has much to do with the efficiency of artificial valves is the procedure employed by culture cells on the scaffolds. In a very recent study, Aleksieva *et al.* have evaluated which culture conditions are optimal for seeding cells onto polyurethane heart valve scaffolds [120]. They compared static cultivation with dynamic cultivation using a conditioning bioreactor. Bioreactors are devices in which biological and/or biochemical processes are manipulated through close control of environmental and process-bound factors such as pH, temperature, pressure, and nutrient and waste flow [121]. After growing endothelial cells and fibroblasts onto the valve scaffolds they evaluated cell confluence, ECM formation and inflammatory response. The study concluded that the use of the bioreactor improved cell attachment to the polyurethane structure and the mechanical properties of the valve scaffold.

In these examples, cell growth is supported *in vitro* and later cell-containing scaffolds are grafted to the animal model. *In situ* tissue engineering represents a new approach in which nude scaffolds are implanted and signaling component presented in the functionalized scaffold guide cell homing, adhesion and growth. The ultimate goal is to achieve complete cellularization of the graft, the production of a new matrix and, finally, tissue formation. Although *in situ* tissue engineering for heart valve reconstruction is an attractive alternative, more studies are needed to elucidate the *in vivo* feasibility of the approach [122].

The second main approach based on nanotechnology directed to improve heart valve diseases conditions is the use of NPs. Atherosclerosis processes that involve heart valve degeneration are the target of functionalized NPs that can be used as a vehicle for drug delivery [123,124,125,126]. In addition, NPs can be used to reduce the risk of thromboembolic events after heart valve surgery [127]. For instance, PLA matrix systems incorporated with PLGA NPs containing nitric oxide donors have been developed for prevention of heart valve complications through sustained and controlled release of nitric oxide [128].

## 8. Clinical Trials

To evaluate the efficacy and safety of nanotechnological devices for application in regenerative therapy it is necessary to perform clinical trials with a large cohort of patients. A clinical trial is conducted through five consecutive phases defined by the FDA [129]. Briefly: (i) Phase 0: Exploratory study involving very limited human exposure to the drug, with no therapeutic or diagnostic goals; (ii) Phase 1: Studies that are usually conducted with healthy volunteers and that emphasize safety; (iii) Phase 2: Studies that gather preliminary data on effectiveness. Safety continues to be evaluated, and short-term adverse events are studied; (iv) Phase 3: Studies that gather more information about safety and effectiveness by studying different populations and different dosages and by using the drug/medical device in combination with other drug/medical device; and, (v) Phase 4: Studies occurring after FDA has approved a drug/medical device for marketing. These studies gather additional information about safety, efficacy, or optimal use.

Here, ClinicalTrials Website [130] was used to look for nanotechnological applications that are currently being evaluated by a clinical trial. The clinical trials we report here have been the result of a search using the terms: nanotechnology, scaffold, devices in conjunction with skin, cartilage, bone, nerve and heart. Table 1 summarized some of the most significant outcomes of our search.

Related to skin tissue, a clinical trial that evaluates the use of *silk sericin scaffold* to wound dressing is being carried out (NCT01539980). Another advanced wound care device that is being clinically tested is *Integra(TM) Flowable Wound Matrix*. Based on collagen technology, this invention provides a scaffold for cellular invasion and capillary growth (NCT01108263). In addition, a multilayer transdermal patch designed by electrospinning, which produces a continuous and stable nitric oxide release, is currently evaluated. The study tries to determine the efficacy of this novel nitric oxide topical donor for the treatment of cutaneous leishmaniasis (NCT00317629). Other clinical trials are evaluating the use of silver NP-coated charcoal dressings with the aim of preventing infection and enhancing recovery in patients with skin burns (NCT01598493, NCT01598480).

In the field of cartilage regeneration, we found a clinical trial that evaluates the regenerative potential of a sponge-like scaffold *BioCart™II* seeded with chondrocytes, to treat cartilage defects of the femoral condyle (NCT00729716). Aa cartilage-repair device called *Kensey Nash Corp* is also being evaluated for clinical use. This device is implanted in the bone below the area of damaged cartilage, the aim of the inventors is that the grafted scaffold will absorb blood and bone marrow from the bone and produce the healing of the damaged cartilage area (NCT01183637).

Interestingly, for bone reconstruction therapy, there is a study that aims to pre-engineer large synthetic bone grafts, to seed then with fat-derived progenitor cells differentiated into osteoblasts and to study the vascularization process *in vivo* (NCT01218945). Another study is a prospective trial to test the safety and feasibility of injectable scaffold (*Ignite^®^)* in combination with progenitor cells (NCT01435434).

An ambitious clinical study that compares different nerve conduit devices for peripheral nerve gap repairs is actually in theirs beginnings. The hollow tube nerve conduits that are going to be evaluated in this trial are Neurotube, NeuroLac, NeuraGen, NeuroMatrix and NeuroFlex (NCT01573650). In addition, we have found a clinical trial that will evaluate nerve repair using a fibrin conduit device to treat patients with traumatic peripheral nerve injury of the finger (NCT01573650).

**Table 1:** Clinical trials on the use of nanotechnological devices for tissue regeneration.

ClinicalTrials.gov Identifier	Clinical trial name	Nanotechnology	Tissue	Status/Phase
NCT01539980	Clinical Study on Silk Sericin Wound Dressing for Split-thickness Skin Graft Donor Sites Treatment	Device: Sericin scaffold	Skin	Phase 1 Phase 2
NCT01108263	Use of INTEGRA™ Flowable Wound Matrix to Manage Diabetic Foot Ulcers	INTEGRA™ Flowable Matrix (Collagen)	Skin	Phase 4
NCT00317629	Controlled Nitric Oxide-Releasing Patch *Versus* Meglumine Antimoniate in the Treatment of Cutaneous Leishmaniasis	Electrospinning-controlled nitric oxide releasing patch	Skin	Phase 3
NCT00729716	Comparison of BioCart™II With Microfracture for Treatment of Cartilage Defects of the Femoral Condyle	BioCart™II scaffold	Cartilage	Phase 2
NCT01183637	Evaluation of an Acellular Osteochondral Graft for Cartilage Lesions Pilot Trial (EAGLE Pilot)	bioresorbable scaffold	Bone/Cartilage	Phase 2
NCT01218945	Development of Bone Grafts Using Adipose-Derived Stem Cells and Different Scaffolds	Bone scaffold	Bone	recruiting participants
NCT01435434	Mononucleotide Autologous Stem Cells and Demineralized Bone Matrix in the Treatment of Non-Union/Delayed Fractures	Ignite^®^ICS injectable scaffold	Bone	Not yet recruiting
NCT00948025	A Comparative Post-Marketing Study of Commercially Available Peripheral Nerve Gap Repair Options (CHANGE)	Device: Hollow tube nerve conduit, synthetic or biosynthetic	Nerve	Active, not recruiting Phase 4
NCT01573650	Optimization of Peripheral Nerve Reconstruction: A Non-Inferiority Trial	Device: Fibrin Conduit	Nerve	not yet open for participant recruitment
NCT01270139	Plasmonic Photothermal Therapy of Flow-Limiting Atherosclerotic Lesions With Silica–Gold Nanoparticles: a First-in-Man Study	Silica–gold nanoparticles. Iron bearing nanoparticles.	Heart	Has results Phase 1–2
NCT00124943	A Phase I/II Safety Trial of Intracoronary Administration of Systemic Nanoparticle Paclitaxel (ABI-007) for the Prevention of In-Stent Restenosis	Nanoparticle paclitaxel	Heart	Has results Phase 1–2

In the field of cardiac tissue regeneration we have not find any data of clinical trials conducted to test heart scaffold devices or synthetic heart valve substitutes. We reported here two clinical trials that evaluate the use of nanoparticules to prevent or treat heart disease-related complications. The first one studies the possible beneficial implications of *silica-gold NPs* in the prevention of atherosclerosis and treatment of its complications (NCT01270139). The second clinical trial evaluates *NP paclitaxel* preliminary efficacy for the prevention of in-stent restenosis (NCT00124943).

## 9. Conclusions

The field of nanotechnology is advancing quickly. This interdisciplinary approach is leading to a rapid expansion and development in the fabrication of biomimetic scaffolds for tissue engineering. Many studies have been conducted in the search for appropriate materials to create a scaffold that may play an active role in the regeneration process instead of simply being a cell carrier or tissue template. The advantages of nanomaterials as therapeutic and diagnostic tools are vast, due to design flexibility, small sizes, large surface-to-volume ratio, and ease of surface modification.

The potential of these bio-devices has shown promising results *in vitro*, and some of them have also been successfully tested *in vivo* with animal models. Nevertheless, the gap between laboratory and medical application of these nanotechnological advances is still wide. Although some successful devises have already being tested in clinical trials and the data produced by these studies is highly encouraging, the safety of nanomedicine is not yet fully defined and more clinical studies still need to be conducted to translate nanotechnological devices to the clinic.

Nanotechnologists, cell biologists and medical doctors have begun to walk the path toward a personalized medicine with the hope of improving the treatment of many diseases. The advanced applications of this approach to regenerative medicine will undoubtedly transform the fundamentals of diagnosis, treatment, and prevention of diseases, becoming an inevitable part of our life.
